# Exploring the Curvilinear Effect of Motivation to Lead on Leadership Emergence: The Moderating Role of Shared Team Vision

**DOI:** 10.3390/bs15101295

**Published:** 2025-09-23

**Authors:** Jinkai Cheng, Yating Luo, Feng Hu, Kunjie Cui

**Affiliations:** 1 Business School, Sichuan University, Chengdu 610065, China; 2 School of Labor and Human Resources, Renmin University of China, Beijing 100872, China; 3 Research Institute of Social Development, Southwestern University of Finance and Economics, Chengdu 611130, China

**Keywords:** motivation to lead, leadership emergence, shared team vision, nonlinear relationship

## Abstract

Given the potential significance of the motivation to lead in answering the question of who will most highly emerge for leadership positions, the motivation to lead has garnered considerable attention. Nevertheless, we put forward a distinct perspective on the influence of the motivation to lead on leadership emergence. Based on the theory of leadership identity construction, we developed and tested a potential curvilinear relationship between individual motivation to lead and leadership emergence while also examining the moderating role of shared team vision. This study involved 639 employees across 159 work teams, with data collected using a multi-wave, round-robin approach. The results of social relations analyses indicated that individual motivation to lead has an inverted U-shaped relationship with leadership emergence. Meanwhile, shared team vision positively moderates the curvilinear relationship, such that those teams with weak shared vision experience foreshortened and weakened positive effect from motivation to lead. These results underscore the importance of comprehending the level of leadership motivation that can promote or prevent leadership emergence within work teams.

## 1. Introduction

How do leaders emerge in self-management work teams? With more and more organizations adopting a team structure to provide employees autonomy and responsibility to achieve organizational goals, this is a research question that has attracted much scholarly attention. Recent years have witnessed a surge of studies on leadership emergence, defined as “the degree to which an individual with no formal status or authority is perceived by one or more team members as exhibiting leaderlike influence” (Hanna et al., 2021, p. 82) (for reviews, see Acton et al., 2019; Badura et al., 2022; Cox et al., 2022). Studies had an increased focus on individual differences such as motivation to lead as predictors of leadership emergence (e.g., Hu et al., 2019; Lemoine et al., 2016; Melwani et al., 2012; Mitchell et al., 2022). According to Badura et al. (2020), motivation to lead represents the desire to attain leadership roles as well as expend effort to fulfill leader role requirements. Because of such individual differences, someone high in motivation to lead should be more likely to enact behaviors within groups aimed at claiming leadership, and as a consequence, be more likely to emerge as leaders within work teams (DeRue & Ashford, 2010).

However, although some studies found that motivation to lead positively predicts leadership emergence (e.g., Badura et al., 2020; Luria & Berson, 2013), others maintained that employees exhibiting high levels of leadership motivation do not necessarily always result in favorable leadership outcomes. For example, Auvinen et al. (2020) found that leaders with high non-calculative motivation to lead experienced poor occupational well-being and were more likely to resign from their current leadership position or apply for less challenging leadership positions. Also, other early work concluded that individuals who have a moderate to high need for power and a low need for affiliation are more likely to emerge into leadership positions (McClelland & Boyatzis, 1982). With this in mind, it is possible that the effect of motivation to lead on leadership emergence might be more complex than would be accounted for by a simple linear regression model.

In this paper, we draw on the leadership identity construction theory (DeRue & Ashford, 2010) to examine the complex relationship between motivation to lead and leadership emergence. This theory views leadership emergence as a process of individuals constructing their leadership identity, which involves three elements: individual internalization—the individual has the desire to attain leadership; relational recognition—others recognize their leadership and are willing to follow; and collective endorsement—individuals are endorsed by the group or social context as exhibiting leaderlike influence. Each element can initiate the leadership identity construction process, and together they facilitate leadership identity construction. This theory thus provides an overarching framework to understand the reciprocal and dynamic nature of leadership identity construction, which has also been adopted by other leadership scholars. For example, Kozlowski et al. (2013) state that leadership emergence does not reside in a person but rather in an interactive dynamic. Likewise, Acton et al. (2019) discuss the importance of multilevel processes (i.e., individual, relational, and collective levels) in the “activation” of leadership emergence. Although no empirical studies have directly examined these processes, indirect evidence suggests that leadership emergence could be predicted by peer reactions and recognition, such as advice seeking by peers and peer liking of the focal person (Hu et al., 2019).

Built on the leadership identity construction theory (DeRue & Ashford, 2010), we study leadership emergence by explicating the individual motivation to lead and social processes and articulating how they independently and jointly contribute to emergent leadership. Specifically, we identify one’s “motivation to lead” as an important individual characteristic that activates individual leadership claiming (Badura et al., 2020). When a person has little motivation to lead, they are unlikely to take the initiative or take on responsibilities, making it difficult for others to recognize their leadership. When a person has a moderate to high motivation to lead, they tend to look for opportunities to demonstrate their willingness to lead and leadership skills by taking initiative, engaging in organizational citizenship behaviors (OCB, Organ, 1990, 1997), etc. Such behaviors are likely attributed as leadership by peers, which facilitates the person’s leadership identity. However, when a person is overly eager to claim leadership, they may engage in aggressive leader-claiming behaviors, such as spreading rumors, slandering others, and forming gangs (S. Sun, 2022), which will make peers fearful and withdraw themselves from making any connections with them. Consequently, peers are cautious to grant these individuals a leader identity, reducing their likelihood of emergence as a leader. Therefore, we argue that a moderate level of motivation to lead is optimal. We predict a curvilinear (inverted U-shaped) relationship between a person’s motivation to lead and their emergence as a leader.

In addition to individual claiming, the social processes revolving around the work team are also important for individual leadership identity construction. In this paper, we identify the social process of the consensus among team members regarding its leadership structure, that is, a shared team schema about leadership. When the consensus is strong, it will be easy for team members to recognize and grant a leader identity to an individual who fits the schema (DeRue & Ashford, 2010). Because a cognitive schema is often hidden and unobservable, we will use shared team vision as a proxy (Pearce & Ensley, 2004) to articulate why this shared vision would mitigate the inverted U-shape relationship predicted earlier.

**Figure 1:**
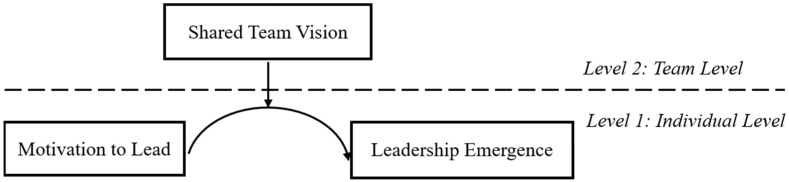
Hypothesized model.

This paper makes two major contributions to the leadership literature by examining the dynamic process of leadership emergence, thus capturing the complexity and interactive nature of the emergent phenomena (Acton et al., 2019; DeRue & Ashford, 2010). First, we provide empirical evidence that individual motivation to lead is not always beneficial for leadership emergence (Badura et al., 2020); rather, their relationship is an inverted U-shape. From a theoretical standpoint, the curvilinear relationship provides direct support to the leadership identity construction theory. Second, our findings suggest that shared team vision moderates the inverted U-shaped relationship between motivation to lead and leadership emergence, which provides a team-level theoretical explanation about when excessive motivation to lead might produce undesired emergent outcomes. In sum, our findings illuminate the social processes involved in emergent leadership. Figure 1 depicts our hypothesized model.

## 2. Theoretical Background and Hypotheses

### 2.1. Motivation to Lead and Leadership Emergence: A Curvilinear Hypothesis

Motivation to lead is defined as individuals’ willingness to engage in leadership training activities and assume leadership roles (K. Y. Chan & Drasgow, 2001), which consists of three components: affective-identity motivation to lead—the degree to which one enjoys leadership roles and sees oneself as a leader, social-normative motivation to lead—the degree to which one views leadership as a responsibility and duty, and non-calculative motivation to lead—the degree to which one views leadership opportunities positively despite potential costs. However, following other scholars’ treatment of this concept (Guillén et al., 2015; Hannah et al., 2012; Schyns et al., 2020), we choose to focus on the affective-identity motivation because it is most closely related to one’s intrinsic motivation to lead, and aligns with a state where individuals come to incorporate the leader identity as part of their self-concept and “claim” their identity as a leader. Moreover, as our research question centers on leadership claiming behaviors’ intensity rather than motivational antecedents, we operationalize motivation to lead as a unitary construct without parsing dimensional differences. This aggregated treatment aligns with our focus on observable outcomes of motivation to lead rather than its psychological underpinnings.

Although previous research has found positive relationships between motivation to lead and leadership emergence (e.g., Elprana et al., 2015; Kennedy et al., 2021; Luria & Berson, 2013), including a recent meta-analysis (Badura et al., 2020), studies also show that people exhibiting high levels of personal proactiveness do not always receive favorable work outcomes (e.g., D. Chan, 2006; Chen et al., 2024; Erdogan & Bauer, 2005; Hu et al., 2019; J. Q. Sun et al., 2021; Wihler et al., 2017). It is possible that the effect of motivation to lead on leadership emergence might be more complex than would be accounted for by simple linear regression models.

Follow the pattern of the “too-much-of-a-good-thing” effect (Pierce & Aguinis, 2013), which is when, after a certain inflection point, a positive influence turns negative, we predict a non-linear relationship between motivation to lead and leadership emergence following an inverted U-shape.

First, when motivation to lead is low, meaning a person lacks the aspiration to lead, it is unlikely for the person to emerge as a leader. In some cases, such a person would explicitly state that they expect someone else to lead and act accordingly. For example, they do not speak in a meeting only when called upon, or actively refrain from taking initiative within the group (DeRue & Ashford, 2010). These behaviors are also likely to be perceived as low willingness to lead by fellow group members, who are unlikely to attribute a leader identity to this person. As a result, the probability for this person to emerge as a leader will be low.

Second, when motivation to lead increases, individuals may look for opportunities to demonstrate their willingness to lead by voluntarily taking on extra role behaviors (OCB, Organ, 1990, 1997) or other initiatives requiring leadership skills. The more they engage in these behaviors, the higher the likelihood that they will internalize a leader identity into their sense of self and claim leadership (Kwok et al., 2018). Consequently, they are likely to be perceived as leader-like by peers (Luria & Berson, 2013).

Third, when the motivation to lead reaches an extremely high level, it can trigger intricate perceptions and emotions among peers. DeRue and Ashford (2010) identified an underlying motive for aspiring to leadership: the pursuit of personal rewards such as promotions, power, status, and a positive reputation (Morgeson et al., 2010), rather than a genuine desire to serve or benefit others (Badura et al., 2020). Consequently, the theory of leadership identity construction posits that the stronger an individual’s perception of the rewards linked to leadership, the more likely they are to claim a leadership identity and assign a follower identity to others. Individuals with a high level of leadership motivation frequently seek to leverage their leadership identity as a tool to acquire power and status. This heightens peers’ sense of threat to their own power and status. As a result, peers may engage in social undermining behaviors to weaken potential competitors, such as deliberately belittling, excluding, or disregarding others’ viewpoints, as well as concealing important information or failing to provide necessary support (Duffy et al., 2012; Vinokur & van Ryn, 1993). Such behaviors restrict the opportunities for these highly motivated individuals to showcase their leadership traits through conventional interpersonal interactions. Ultimately, it obstructs their ability to gain endorsement from peers as a leader.

Particularly, an entrenched philosophy of life in Chinese culture, *Zhong Yong* has an inevitable impact on how people in this culture behave. It encourages people to adopt a moderate rather than an extreme approach when dealing with various matters (Yao et al., 2010). Indeed, a study of samples from Chinese employees has shown that assertiveness has an inverted U-shaped relationship with leadership emergence (Hu et al., 2019), suggesting that a moderate level of positive leadership attributes is even more likely to draw peers’ leadership granting. Similarly, in this study, we propose that the relationship between MTL and leadership emergence might be curvilinear, where motivation to lead positively influences leadership emergence up to an optimal intermediate level, but beyond this, the relationship tapers off, becoming flat and eventually negative. We therefore propose the following hypothesis:

**Hypothesis** **1.** *Motivation to lead has an inverted U-shaped relationship with leadership emergence, such that a moderate level of motivation to lead results in the highest level of leadership emergence.*

### 2.2. The Moderating Role of Shared Team Vision

As noted by DeRue and Ashford (2010), the context in which the claiming and granting of leadership is important. Specifically, in the context of a shared schema where the boundaries between a leader’s identity and a follower’s identity are permeable, there would be few identity conflicts and little tension over leadership (DeRue & Ashford, 2010). It thus follows that a shared team vision may serve as a moderator that could effectively influence the level of inflection point of the inverted U-shape relationship between individual motivation to lead and emergent leadership. Shared team vision refers to “a common mental model of the future state of the team or its tasks that provides the basis for action within the team” (Pearce & Ensley, 2004). A shared team vision can help build a supportive climate that facilitates informal leadership emergence (Zhang et al., 2012) because it provides clarity and visibility about what team members are striving to achieve, which will guide the actions of individuals willing to claim leadership, and peer assessment of their leadership quality related to the leadership granting process (DeRue & Ashford, 2010). When team members share a common vision, there will be great alignment between the team’s sense of purpose and individuals’ leadership claiming and peers’ leadership granting. Therefore, for teams with a high degree of shared vision, the inflection point of the inverted U-shape relationship between motivation to lead and leadership emergence will be high.

In contrast, when team vision sharedness is low, members are likely to pursue their own goals without a common sense of purpose (Zhang et al., 2012). In this case, the clarity and credibility of individual actions are low, leaving room for interpretation about whether certain leadership claiming behavior is motivated by selfish reasons, which casts doubts on peer evaluation of true intentions. As a result, peers are less likely to grant leadership to these individuals. Taking together the above arguments, we propose the following hypothesis:

**Hypothesis** **2.** *Shared team vision moderates the curvilinear relationship between motivation to lead and leadership emergence, such that a higher inflection point occurs on the inverted-U shape for teams characterized by high shared vision (2a) and a lower inflection point occurs on the inverted-U shape for teams characterized by low shared vision (2b).*

## 3. Method

### 3.1. Participants and Procedure

We collected our data from 733 full-time employees across 180 teams working in a major automobile manufacturer in China that specializes in developing, producing, and selling commercial vehicles, such as sedans, pickup and pump trucks, agitating lorries, and cranes. The selected teams were engaged in various organizational functions, including comprehensive management (37.1%), marketing and sales (20.8%), and research and development (11.3%). We believe that our sample is diverse enough for testing our proposed model. Specifically, the selected teams are based in different cities across China, such as Beijing, Changsha, Zhucheng, Foshan, and Weifang, thus allowing us to evaluate the generalizability of our conclusions (Ali et al., 2020). All of these teams are completely self-managed, and each team enjoys a high degree of interdependence, as evidenced by how team members coordinate with one another when planning and performing their tasks. Moreover, all employees within each team are working at the same rank level. These characteristics offer an ideal context for investigating the interplay between team members and leadership emergence.

We started recruiting our participants after the corresponding author obtained permission from an executive contact within the selected company. We distributed online questionnaire survey links to the participants with the help of the HR department of each company subsidiary. Instructions included a consent form notifying respondents that completing the survey was voluntary and that we would keep their responses strictly confidential and only use these data for research purposes. To minimize issues related to common method bias, we adopted a three-wave, round-robin research design (Podsakoff et al., 2012). Specifically, during Time 1 (T1), we invited 733 employees from 180 teams to answer questions related to their demographics (i.e., gender, age, and educational achievement), job tenure, and motivation to lead. Three months later, during Time 2 (T2), we invited the same employees to evaluate the shared vision within their teams. Six months later, during Time 3 (T3), we evaluated the leadership emergence of each participant as perceived by his/her peers using the round-robin approach.

We further refined our sample by excluding those participants who failed the quality checks (adding bogus items to the questionnaires) or showed no variance in their responses (Dunn et al., 2018). We also eliminated those teams where only one or two members completed the questionnaire. We ultimately retained 639 complete responses from 159 teams, yielding an 87.2% final response rate. Each selected team had an average of 3 to 6 participants, with an average of 4.02 participants (*SD* = 0.85). Among these participants, 50.7% were male, 86.7% held a college degree, and 77.9% had been working at the company for over 5 years.

### 3.2. Measures

We used Chinese to administer our measures. Following Brislin (1970), the first author translated the items into Chinese, and then the second author back-translated these translations into English. All authors thoroughly reviewed the original and back-translated items and amended any discrepancies to guarantee conceptual similarity. We used a five-point Likert scale (1 = *strongly disagree* to 5 = *strongly agree*) to measure all items except when specified otherwise.

**Motivation to lead (MTL).** Following previous studies, we used the nine-item scale proposed by K. Y. Chan and Drasgow (2001) to measure MTL. This same scale has also been adopted in the literature (e.g., Kennedy et al., 2021; Luria & Berson, 2013; Schyns et al., 2020). Some sample items include “Most of the time, I prefer being a leader rather than a follower when working in a group,” “I am the type of person who likes to be in charge of others,” and “I have a tendency to take charge in most groups or teams.” This scale obtained an internal consistency of *α* = 0.941.

**Leadership emergence.** We used the single-item scale proposed by Carson et al. (2007) to measure leadership emergence. This scale has also been used to measure leadership emergence in several studies (e.g., Liu et al., 2018; Melwani et al., 2012; Zhang et al., 2012). Specifically, we asked the participants to rate each of their teammates in terms of their team leadership qualities on a scale of 1 (*not at all*) to 5 (*to a great extent*). We obtained the following peer agreement and reliability values, which generally support our choice to use these ratings to represent leadership emergence: interrater agreement, median r_WG_ = 0.71; interclass correlation, ICC (1) = 0.32; reliability of group mean, ICC (2) = 0.60; and between-group variance, value of F (638, 1403) = 2.47, *p* < 0.001 (Biemann et al., 2012; James et al., 1984). We obtained ICC (2) values below the acceptable threshold of 0.70, but some teams included in our sample only had 3 to 6 members. Previous studies (Bliese, 1998; Gong et al., 2009) suggest that teams with less than 10 members limit the use of ICC (2) for justifying data aggregation, but low ICC (2) values may still be accepted if high r_WG_ and ICC (1) values are present along with significant F statistics (Ali et al., 2020; Dietz et al., 2015). Therefore, the results above support our individual-level aggregation of the data.

**Shared team vision.** We measured shared team vision using the three-item scale proposed by Pearce and Ensley (2004), which was used in Zhang et al. (2012). We asked multiple team members to rate this team-level construct. Sample items include “My teammates provide a clear vision of where our team is going,” “My teammates provide a clear vision of who and what our team is,” and “Because of my teammates, I have a clear vision of our team’s purpose.” We then aggregated these individual responses to generate a team-level score, a decision that was supported by the high interrater agreement, high ICCs, and significant F statistic (median r_WG(J)_ = 0.89, ICC (1) = 0.15, ICC (2) = 0.42, F (158, 479) = 1.72, *p* < 0.001). This scale obtained Cronbach’s alphas of 0.913 and 0.938 at the individual and team levels, respectively.

**Control variables.** We included several controls to account for alternative explanations of leadership emergence. First, we regarded the demographic characteristics of the team members, including their gender (1 = *male*, 0 = *female*), age, and education, and several work-related variables, including job tenure, as control variables given that these variables reflect individual and work differences that may impact leadership emergence (Chiu et al., 2016; Hanna et al., 2021; Lemoine et al., 2016; Zhu et al., 2018). Second, we controlled for team size (mean = 4.02, SD = 0.85) to address potential alternative explanations and ensure a conservative team-level evaluation of our model (Hanna et al., 2021). Third, some studies (e.g., Acton et al., 2019; Badura et al., 2020; Hannah et al., 2008; Kwok et al., 2018) reveal an association between leadership self-efficacy and leadership emergence. Therefore, we measured the participants’ leadership self-efficacy (measured in the second survey) by adapting the eight-item scale proposed by Kane and Baltes (1998) that was applied in several studies (e.g., Guillén et al., 2015; McCormick et al., 2002). We then included leadership self-efficacy as a control variable to eliminate its effects on leadership emergence. This variable obtained a reliability of *α* = 0.941. Finally, some studies (e.g., Charlier et al., 2016; Purvanova et al., 2021) underscore the importance of virtuality context in predicting leadership emergence. Accordingly, we controlled for team virtuality (measured in the second survey), which describes “the extent to which team members use virtual tools to coordinate and execute team processes, the amount of informational value provided by such tools, and the synchronicity of team member virtual interaction” (Kirkman & Mathieu, 2005). To measure team virtuality, we adapted the six-item scale proposed by Chudoba et al. (2005) and asked the participants to rate their experiences on the following items from 1 (*not at all*) to 5 (*to a great extent*): “collaborate with people who have never met face to face,” “work at different sites,” and “work at home during normal business days.” We then aggregated the individual responses to generate a team-level score, a decision that was supported by the high interrater agreement, high ICC, and significant F statistic (median r_WG(J)_ = 0.80, ICC (1) = 0.22, ICC (2) = 0.53, F (158, 479) = 2.15, *p* < 0.001). This scale obtained Cronbach’s alphas of 0.832 and 0.888 at the individual and team levels, respectively.

## 4. Results

### 4.1. Confirmatory Factor Analyses and Descriptive Statistics

We used Mplus version 8.3 to test our hypotheses. We tested our measurement model beforehand by conducting Multilevel CFA (i.e., MTL, shared team vision, leadership self-efficacy, and team virtuality) while excluding the single-item measures (i.e., leadership emergence). Results from Table 1 confirmed the adequate fit of the four-factor model (*χ*^2^ = 535.58, *df* = 144, *p* < 0.001, RMSEA = 0.07, CFI = 0.95, TLI = 0.94, SRMR-_individual_ = 0.03, SRMR-_team_ = 0.07). This model also had a better fit than any of the alternative models when loading any pair of variables on a single factor, thereby suggesting that common method variance did not significantly influence our results.

Table 2 displays the means, standard deviations, and correlations of the descriptive variables at the individual and team levels. Results highlighted a significant relationship between MTL and leadership emergence (*r* = 0.21, *p* < 0.01).

### 4.2. Analysis and Results

**Figure 2:**
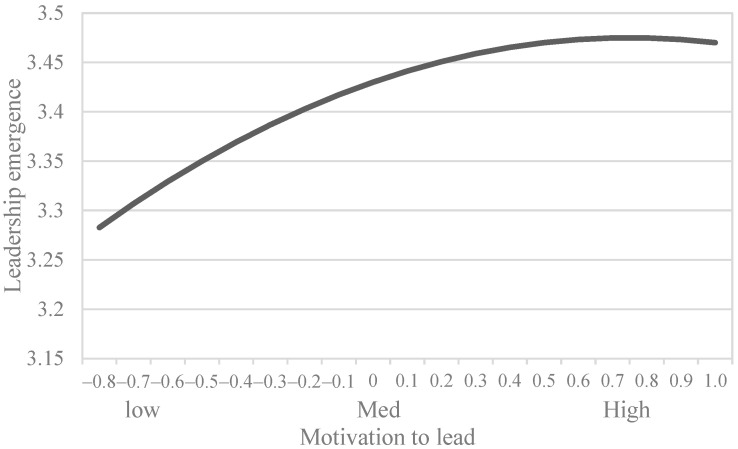
Curvilinear relationship between MTL and leadership emergence.

We performed multilevel regression modeling to estimate the curvilinear effect of MTL on leadership emergence and to examine the moderating effect (Table 3). H1 proposed that MTL exerts a curvilinear effect on peer-rated leadership emergence. For a curvilinear relationship Y = b_0_ + b_1_ × X + b_2_ × X^2^ + e, the inflection point was calculated as: −b_1_/(2 × b_2_). In Table 3, we initially estimated a model with leadership emergence as a control variable (Model 1). We tested H1 by adding MTL and its squared term (MTL-squared) as predictors and shared team vision as a moderator to Model 1. We also group-mean-centered MTL before computing MTL-squared to reduce multicollinearity (Aiken & West, 1991). Model 2 in Table 3 showed that despite the positive relationship between MTL and leadership emergence (*b* = 0.12, *SE* = 0.05, *p* < 0.01), MTL-squared had a negative coefficient (*b* = −0.08, *SE* = 0.04, *p* < 0.05), indicating an inverted U-shaped curve with a positive trend (Li et al., 2018). We plotted this curve in Figure 2 following the procedures recommended by Cohen et al. (2003). As shown in this figure, MTL had an inverted U-shaped relationship with leadership emergence, and this relationship demonstrated upward and downward trends at low and high levels of MTL, respectively. The inflection point was calculated as 0.75 (i.e., −b_1_/(2 × b_2_) = −0.12/(2 × (−0.08)), thereby supporting H1.

**Figure 3:**
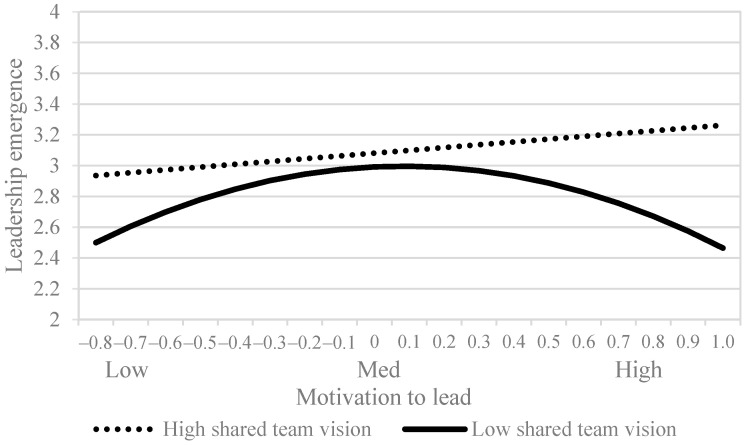
Moderation effect of shared team vision on the relationship between MTL and leadership emergence.

H2 proposed that shared team vision moderates the curvilinear effect of MTL on leadership emergence. We followed the recommendations of Pierce and Aguinis (2013, p. 326, Equation (4)) and tested the moderated curvilinear relationship using the following equation:

Y = b_0_ + b_1_ × X + b_2_ × M + b_3_ × XM + b_4_ × X^2^ + e,

where X was the predictor (i.e., MTL), M was the moderator (i.e., shared team vision), and Y was the outcome variable (i.e., leadership emergence). In this model, when the moderator was at high versus low levels (M_H_ and M_L_), the inflection points were −(b_1_ + b_3_ × M_H_)/(2 × b_4_) and −(b_1_ + b_3_ × M_L_)/(2 × b_4_), respectively. The shift of the inflection points was the difference between these two values. Previous studies (Hu et al., 2019; Li et al., 2018) used the same approach to test moderated curvilinear effects. As shown in Table 3, we added the interaction terms between MTL and shared team vision and between MTL-squared and shared team vision into Model 2 to generate a moderation model (Model 3) for testing this hypothesis. We then obtained unbiased estimates of the cross-level interaction effects by group- and grand-mean centering MTL and shared team vision, respectively (Hofmann & Gavin, 1998). Model 3 in Table 3 showed a significant interaction term between MTL-squared and shared team vision (*b* = 1.32, *SE* = 0.58, *p* < 0.05). We then tested the linear slopes and curvatures for the regressions predicting leadership emergence to further illustrate the moderating influence of shared team vision on the nonlinear relationship between MTL and leadership emergence. We examined these slopes and curvatures at low (−1 SD) and high (+1 SD) levels of shared team vision, and results showed a significant and positive slope term (slope = 0.18, *SE* = 0.05, *p* < 0.01) and an insignificant curvature term for teams with high shared vision (curvature = 0.47, *SE* = 0.26, *ns*). These results confirmed the positive linear relationship of MTL with leadership emergence in the presence of high shared team vision, thereby rejecting H2a. Meanwhile, we obtained an insignificant slope term (slope = 0.11, *SE* = 0.69, *ns*) and a significant and negative curvature term for teams with low shared vision (curvature = −0.63, *SE* = 0.23, *p* < 0.01), which suggested an inverted U-shaped relationship between MTL and leadership emergence in the presence of low shared team vision. Figure 3 plots this moderating effect and suggests that for teams with lower shared vision, the inverted U-shaped relationship between MTL and leadership emergence had a lower inflection point. The inflection point was calculated as 0.67 (i.e., −(b_1_ + b_3_ × M_L_)/(2 × b_4_) = −(0.12 + 0.03 × (−0.42))/(2 × (−0.08)), thereby supporting H2b.

## 5. Discussion

We contribute to the leadership identity construction theory by identifying MTL as a precursor to leadership emergence and examining the moderating influence of shared team vision on this relationship. Our research findings stand in contrast to the conclusions of prior MTL studies (Badura et al., 2020). Specifically, we demonstrate that MTL has a curvilinear impact on leadership emergence. When MTL reaches an excessively high level, its marginal contribution to leadership emergence begins to decline, resulting in diminishing returns. Shared team vision acts as a crucial boundary condition in this relationship. Teams with a weak shared vision experience a more limited and less potent positive effect from MTL, indicating that the effectiveness of MTL is closely tied to the strength of the team’s shared vision.

### 5.1. Theoretical Implications

Our findings above offer several key contributions to theory. First, these findings present a comprehensive and well-nuanced comprehension of the impacts of MTL on leadership emergence within organizational settings. Prior research predominantly portrays MTL as a significant predictor of leadership emergence (Badura et al., 2020; Elprana et al., 2015; Hong et al., 2011; Luria & Berson, 2013). They place strong emphasis on the linear advantages of MTL for work teams. In these teams, members who display a keen interest and strong desire to take on leadership roles are anticipated to become either formal or informal leaders (Badura et al., 2022). Building on but deviating from these earlier studies, our research delves into the potential adverse effects of a high level of MTL. This research avenue aligns with the recent trend in organizational research, which focuses on uncovering the negative impacts of positive individual traits. Examples of such traits include helping motivation (Lin et al., 2019), proactive personality (J. Q. Sun et al., 2021), and extraversion (Hu et al., 2019). As DeRue and Ashford (2010) pointed out, “the construction of a leadership identity occurs when claims and grants of leader identity are endorsed” (p. 633). Our findings further reinforce the proposition of leadership identity construction theory. That is, an individual’s assertion of a leadership role does not necessarily result in the actual conferment of that role if their peers do not support this claim.

Secondly, we further emphasize that a shared team vision serves as a crucial boundary condition for leadership emergence. While numerous studies have accentuated the cross-level impact of a shared team vision on leadership emergence (e.g., Zhang et al., 2012), only a handful of research endeavors have delved into how the motive to lead (MTL) and a shared team vision collectively forecast leadership outcomes. Our findings indicate that individuals with a strong aspiration to lead are less prone to assume leadership positions when their team members possess a low level of shared vision. By examining the boundary conditions of MTL through the lens of contextual factors, we also heed Badura et al.’s (2020) call for more in-depth research on MTL within the organizational context.

Third, this research advances leadership identity theory by proposing a dynamic fit mechanism between individual motivation and team cognition (DeRue & Ashford, 2010). Through social relations modeling, we demonstrate that leadership emergence occurs when an individual’s MTL optimally aligns with the team’s shared vision. The inverted U-curve reflects a “motivation–cognition” mismatch phenomenon: both excessively high MTL (perceived as threatening to team cohesion) and insufficient MTL (lacking initiative) disrupt the identity construction process. This theoretical contribution elucidates how leadership identities are co-constructed through the interplay of personal drive and collective cognitive evaluation, enriching our understanding of the contextual boundaries for effective leader emergence (Acton et al., 2019; Cox et al., 2022). The findings establish shared vision as a critical calibrator that determines the functional range of MTL in team settings.

### 5.2. Practical Implications

Our research findings present significant implications that can effectively guide both organizations and employees in the processes of selecting informal leaders and designing training programs.

Firstly, our results caution organizations that a high level of MTL may have a “too-much-of-a-good-thing” effect on leadership emergence. Individuals with moderate MTL levels typically exhibit amiable behavior in their social interactions. They do not over-dominate conversations or interactions, which enables them to easily win the approval of their colleagues. In contrast, those who assertively seek leadership positions often have the opposite effect. Therefore, organizations need to carefully weigh the pros and cons of recruiting individuals with intense leadership aspirations. To illustrate, during the probationary period, a comprehensive 360 degree assessment can be employed. This assessment should focus on evaluating a candidate’s leadership motivation as well as their likeability among peers. Such an evaluation will assist managers in accurately identifying and selecting the most suitable candidates. Managers are advised to choose and integrate new team members who possess a moderate level of leadership motivation and are well-received by their colleagues. Moreover, managers should devise strategies to support employees with moderate MTL. This can involve implementing targeted training programs to enhance their leadership skills, offering constructive and positive feedback on the impact they are making within the organization, and strengthening their self-perception as effective leaders. Through these measures, employees with moderate MTL can be better equipped to contribute to the organization’s leadership development.

Second, our research findings emphasize the crucial role of a shared team vision in the transition from MTL to leadership emergence. Managers need to recognize that leadership is highly unlikely to emerge in teams with a weak shared vision. To tackle this issue, managers can arrange workshops where team members can work on cultivating shared team values and a common vision (Zhang et al., 2012). Additionally, managers should be cognizant that excessive leadership aspirations in the absence of a shared team vision can be perceived negatively during team interactions. Consequently, management should train team members to be more receptive to others’ ideas and more mindful of others’ interests, which will facilitate the development of a shared vision within the team.

Third, our findings suggest that both relational recognition and individual leadership internalization are necessary simultaneously in the process of leadership emergence. Team leadership is regarded as a collective asset, where every team member, regardless of their job position or role, has the potential to lead (DeRue & Ashford, 2010; Zhu et al., 2018). Therefore, organizations aiming for shared leadership should place greater emphasis on the social and interactive process mechanisms that underpin leadership emergence.

### 5.3. Limitations and Future Research Directions

Our research is subject to several limitations that should be taken into account when interpreting the results. Firstly, this study was conducted within a single organization—a motor company—with teams spread across China. China is characterized by high levels of collectivism, power distance, and the *Zhong Yong* value system (Bachrach et al., 2007; Farh et al., 2007). These cultural and organizational features may undermine the generalizability of our findings. To address this issue, future research could draw samples from diverse industries and countries. Additionally, while we employed the round-robin approach and a time-lagged design to collect data on our variables, future studies should explore alternative measurement methods, such as experience sampling. This would enable a more comprehensive understanding of the dynamic and complex nature of leadership emergence and provide additional validation for our results.

Secondly, our study relied solely on concepts from leadership identity construction theory, specifically the MTL concept, to explain leadership emergence within organizations. We assumed a consistent alignment between MTL and leadership-claiming behaviors. However, future research should directly measure individual leadership-claiming behaviors and peer responses. For instance, as Hu et al. (2019) pointed out, individuals who exhibit relationship-based behaviors are more likely to be perceived as leader-like and selected as leaders. Conversely, overly aggressive leadership-claiming behaviors may instill fear in peers, hindering the emergence of leadership.

Thirdly, this study’s concentration on MTL intensity neglects critical variations across affective, calculative, and social-normative motivations (K. Y. Chan & Drasgow, 2001), potentially masking their divergent effects on leadership emergence. Each dimension distinctly shapes assertive behaviors and peer evaluations (e.g., affective drives enhancing charisma versus calculative motives provoking distrust). Future investigations should employ a multidimensional approach to dissect how each MTL category distinctly operates, probing their differential linkages to emergent leadership. Such an advancement would elucidate whether MTL’s dimensional contrasts (beyond mere intensity) more effectively account for central leadership emergence barriers.

Lastly, despite focusing on the shared team vision and controlling for team virtuality, our study did not fully account for other contextual factors that can influence informal leadership emergence. These factors include power distance, gender egalitarianism, social networks, and personality differences. Therefore, we emphasize the importance of incorporating such contextual factors in future leadership emergence research. These factors can potentially enhance an individual’s emergent leadership by creating opportunities for incremental leadership successes.

## 6. Conclusions

From a multilevel perspective, we delved into the antecedents of leadership emergence, taking into account both individual leadership motivation and the shared vision within the team. Moreover, we combined the assertions and bestowals of leadership identity to comprehensively understand the process of leadership emergence. An individual who exhibits a moderate level of MTL and upholds a shared vision with fellow team members stands a greater chance of being entrusted with a leadership position within the team. We anticipate that our research findings will serve as a catalyst for further exploration into the intricate social processes that underpin the relationship between MTL and leadership emergence.

## Figures and Tables

**Table 1:** Multilevel confirmatory factor analysis for the full measurement model.

Factor Model	# of Factors	*χ* ^2^	*df*	RMSEA	CFI	TLI	SRMR-_individual_	SRMR-_team_
Full measurement model	4	535.576	144	0.065	0.950	0.940	0.028	0.070
Shared team vision and team virtuality collapsed (a)	3	837.135	145	0.086	0.911	0.895	0.028	0.161
MTL and leadership self-efficacy collapsed (b)	3	3831.998	145	0.199	0.527	0.438	0.253	0.070
a and b	2	4170.047	146	0.208	0.483	0.391	0.253	0.161

Note. N = 639 individuals within 159 teams. MTL = motivation to lead.

**Table 2:** Means, standard deviations, and correlations among study variables.

	Mean	SD	1	2	3	4	5	6	7	8	9	10
**Level 1: Individual (N = 639)**												
1. Gender (T1)	0.51	0.50										
2. Age (T1)	1.96	0.77	0.01									
3. Education (T1)	2.01	0.53	−0.10 **	−0.15 **								
4. Job tenure (T1)	2.77	1.33	0.03	0.86 **	−0.27 **							
5. Leadership self-efficacy (T2)	4.12	0.63	0.15 **	0.01	−0.01	0.07	(0.94)					
6. MTL (T1)	3.42	0.80	0.30 **	−0.07	0.09 *	−0.03	0.28 **	(0.94)				
7. Leadership emergence (T3)	3.45	0.82	0.12 **	0.06	0.10 *	0.10 *	0.18 **	0.21 **				
**Level 2: Team (N = 159)**												
8. Team size	4.20	0.85	0.09 *	0.05	−0.07	0.07	0.02	−0.01	−0.03		−0.06	0.02
9. Team virtuality (T2)	3.23	0.53	0.24 **	0.04	0.04	0.02	0.36 **	0.20 **	0.15 **	−0.03	(0.89)	0.38 **
10. Shared team vision (T2)	4.09	0.42	0.11 **	−0.02	0.03	0.02	0.54 **	0.18 **	0.15 **	0.03	0.30 **	(0.94)

Note. Correlations below the diagonal represent individual-level (Level 1) correlations (N = 639). Correlations above the diagonal represent team-level (Level 2) correlations (N = 159). Cronbach’s alpha coefficients are presented in the parentheses. SD = standard deviation; T1/2/3 = time 1/2/3; gender is coded 1 = male, 0 = female; age is coded 1 = 20–30 years old, 2 = 31–40 years old, 3 = 41–50 years old, 4 = 51–60 years old; education is coded 1 = high school degree, 2 = bachelor’s degree, 3 = master’s degree, 4 = doctoral degree; job tenure is coded 1 = less than 5 years, 2 = 6–10 years, 3 = 11–15 years, 4 = 16–20 years, 5 = above 20 years. Team size is coded 3 = 3 people, 4 = 4 people, 5 = 5 people, 6 = 6 people. * *p* < 0.05; ** *p* < 0.01 (two-tailed).

**Table 3:** Multilevel regression modeling results.

	Leadership Emergence					
Variable	Model 1		Model 2		Model 3	
	Estimate	B (*SE*)	Estimate	B (*SE*)	Estimate	B (*SE*)
**Level 1: Individual (N = 639)**						
Intercept	3.46 **	2.70 (0.03)	3.43 **	83.00 (0.04)	3.42 **	83.04 (0.04)
Gender	0.11	1.50 (0.07)	0.05	0.68 (0.07)	0.05	0.68 (0.07)
Age	−0.07	−0.89 (0.08)	−0.07	−0.81 (0.08)	−0.07	−0.81 (0.08)
Education	0.24 **	3.63 (0.07)	0.21 **	2.95 (0.07)	0.21 **	2.95 (0.07)
Job tenure	0.16 **	3.06 (0.05)	0.15 **	3.00 (0.05)	0.15 **	3.00 (0.05)
Leadership self-efficacy	0.16 **	2.93 (0.06)	0.14 *	2.50 (0.06)	0.14 *	2.51 (0.06)
Motivation to lead (MTL)			0.12 **	2.70 (0.05)	0.12 **	2.70 (0.05)
MTL^2^			−0.08 *	−1.98 (0.04)	−0.08	−1.81 (0.04)
**Level 2: Team (N = 159)**						
Team size	−0.02	−0.44 (0.04)	−0.03	−0.80 (0.04)	−0.00	−0.04 (0.04)
Team virtuality	0.36 **	5.19 (0.07)	0.27 **	3.48 (0.08)	0.24 **	3.01 (0.08)
Shared team vision (STV)			0.30 **	3.00 (0.10)	1.02 **	2.98 (0.34)
**Cross-level interactions**						
MTL × STV					0.03	−0.02 (1.63)
MTL^2^ × STV					1.32 *	2.28 (0.58)
Pseudo R^2^ _Level 1_	0.055 **		0.069 **		0.069 **	
Pseudo R^2^ _Level 2_	0.349 **		0.479 **		0.598 **	

Note. N = 639 employees within 159 teams. Unstandardized path coefficients are reported in the table. *SE* = standard error. * *p* < 0.05; ** *p* < 0.01 (two-tailed).

## Data Availability

The data that support the findings of this study are available from the corresponding author upon reasonable request.
